# Progress in Pediatrics

**Published:** 1902-10-25

**Authors:** 


### PROGRESS IN PEDIATRICS.

(*Concluded from page* 51.)

**Acute Rheumatic Endocarditis with a Congenital Heart-lesion.**—Halle and Armand Delille[14] report the case of a boy, aged three years, who developed an attack of acute articular rheumatism. The cardiac action was rapid and heaving, and a systolic murmur was present at the apex, transmitted to the back. Death followed from cardiac failure after several hours of severe progressive dyspnœa and cyanosis. At the autopsy the mitral valve was much thickened and presented fresh vegetations, while the foramen ovale was open and as large as a franc-piece. This case is remarkable for the previous absence of cyanosis or any functional disturbance due to the congenital defect, and for the rapidity with which the fatal termination ensued. It thus exemplifies the rule recently formulated by Marfau: whenever an acquired endocarditis in childhood presents very grave symptoms it is probably accompanied either by pericarditis or a congenital defect of the heart.

**Post-rectal Dermoid Tumour containing True Bone.**—A case of this nature is reported by Dr. Sinclair White[15] in a girl of three years. At birth a tumour as large as a walnut was present behind the anus. This had gradually increased in size, but produced no discomfort until two months previously, when the child fell on her buttocks. After the fall the tumour increased rapidly in size, and began to be painful. The whole mass was dissected out easily, and proved to be a dermoid cyst, containing pus, sebaceous material, hairs, and incisor teeth, growing from a piece of bone as large as a patella. Microscopic examination of the bone showed the presence of Haversian canals. It has been usually held that a true dermoid tumour contains only epithelial tissues, but this specimen shows that the boundary-line between dermoids and teratomata is not a hard and fast one.

**Examination of the Gastric Contents.**—An investigation into this subject has been made by Dr. Louis Fisher,[16] who describes fully the methods he employed. In atrophic children and in subacute dyspeptic conditions HCl could not be found. At the beginning of the digestive process lactic acid predominates, and at the end of digestion hydrochloric acid is in excess. The gastric contents of children fed on raw milk warmed to 100° F. always showed a better state of digestion than those of children fed on superheated milk. Cheesy curds in hard leathery masses were found two to three hours after a meal of sterilised milk, and on the substitution of raw milk these were replaced by more flocculent and less thick curds.

**Infantile Scurvy.**—Dr. George Carpenter[17] considers that, while undoubtedly a faulty diet is the cause of this affection, there is also some unknown personal factor to be considered. For several members of a family may be suitably reared on a diet which proves eminently scorbutic to another infantile member of that family. Even infants at the breast and newly born children have developed scurvy, although such occurrences are rare. Certain foods are especially noxious to certain children, and of these harmful foods the proprietary foods hold the first place. He relates the case of a rickety boy, five years old, who developed scurvy of the infantile type. Simple hæmaturia and simple purpura, when occurring in children after infancy, are often due to a defective diet and essentially scorbutic in origin, even although no gingival or other characteristic signs of scurvy are present.

[14] Arch. de Med. des Enf., 1901, Vol. IV., No. 5, and Amer. Jour. Med. Sci., Nov., 1901. [15] Quart. Med. Jour., Nov., 1901, p. 55. [16] Med. News, July 5, 1902, p. 13. [17] Lancet, May 3, 1902, p. 1246.

